# Anisotropic Hot Spot Formation at a Grain Boundary in Shock-Compressed TATB High Explosive Crystal

**DOI:** 10.1021/acs.jpcc.6c00496

**Published:** 2026-04-14

**Authors:** Matthew P. Kroonblawd, Nithin Mathew, Puhan Zhao, Shan Jiang, Edward M. Kober, Tommy Sewell

**Affiliations:** † Physical and Life Sciences Directorate, 4578 Lawrence Livermore National Laboratory, Livermore, California 94550, United States; ‡ X Computational Physics Division, 5112 Los Alamos National Laboratory, Los Alamos, New Mexico 87545, United States; § Center for Clinical Pharmacology, Washington University School of Medicine and University of Health Sciences and Pharmacy, St. Louis, Missouri 63110, United States; ∥ Department of Anesthesiology, School of Medicine, Washington University in St. Louis, St. Louis, Missouri 63110, United States; ⊥ Department of Mechanical Engineering, 8083 University of Mississippi, University, Mississippi 38677, United States; # Theoretical Division, 5112 Los Alamos National Laboratory, Los Alamos, New Mexico 87545, United States; ∇ Department of Chemistry, 14716 University of Missouri, Columbia, Missouri 65211, United States; ○ Materials Science and Engineering Institute, 14716 University of Missouri, Columbia, Missouri 65211, United States

## Abstract

Secondary high explosives (HEs) exhibit rich microstructure that promotes the formation of hot spots responsible for detonation initiation, but the role of microstructural interfaces remains poorly quantified. To this end, we develop extensions for the generalized crystal-cutting method (GCCM) to prepare molecular dynamics (MD) simulation cells containing grain boundaries (GBs) and other crystal–crystal interfaces with prescribed tilt and twist orientations. Using the GCCM, we perform MD simulations of shock interactions with a GB between the (001) and (100) crystal facets in the secondary HE TATB (1,3,5-triamino-2,4,6-trinitrobenzene). Our MD simulations reveal a strong directional dependence to the formation of a hot spot at the GB interface. In particular, transmission of the shock from the (001) grain to the (100) grain yields a hot spot in the (100) grain at the GB interface, whereas no hot spot is produced when an equivalent shock transits the GB in the opposite direction. We trace the origin of this GB anisotropy to three dominant factors: (1) the intrinsic differences in shock-deformation mechanisms and wave structures for the bulk (100) and (001) grains, which leads to distinct geometries and mechanical impedances upon shock arrival to the GB depending on which grains are donor or acceptor for the transmitted shock; (2) the different time intervals separating the initial shock rise and the formation of steady wave structures in the respective donor–acceptor configurations; and (3) the differences in time scales required to re-establish local thermal equilibrium. Interfacial hot spots form when these factors combine to impede development of a steady two-wave structure and instead induce a localized, pseudosingly shocked region that undergoes a higher rate of work production (resulting in a higher temperature) compared to when the steady two-wave structure develops further from the interface. The extensions to the GCCM approach presented here are anticipated to facilitate a wide range of MD studies that focus on understanding the role of crystal–crystal interfaces in molecular materials.

## Graphical abstract

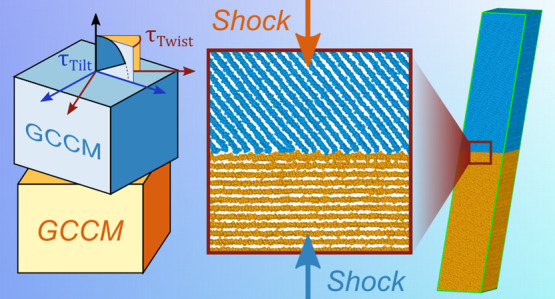

## 1 Introduction

Formation of hot spots at microstructural defects is understood to control the detonation initiation behavior of organic secondary high explosives (HEs).^1^ A hot spot is formed when a mechanical impulse (e.g., a shock wave) interacts with microstructural features to localize dissipation resulting in isolated elevated temperatures that accelerate chemical reactions. If the temperature rise is sufficient to induce chemical reactions, this hot spot can ignite the locally heated material and potentially grow if exothermic reactions consume the surrounding material.^2–4^ Multiple hot spot formation mechanisms are known,^5,6^ but shock-induced pore collapse is widely understood to be the primary source for hot spots that control initiation of secondary HEs under strong shock loading.^1^ (Comparatively slow collapse of pores under weak shock loading can squeeze out the porosity without producing enough heat to ignite chemistry; the resulting HE becomes desensitized, even when subjected to shock pressures that are much higher than the normal initiation threshold.^7^) Hot spot formation via pore collapse has been extensively studied in a wide variety of organic HEs, but less attention has been given to characterizing hot spot formation at other kinds of microstructural defects such as interfaces. This is perhaps surprising because most of the HEs in use are formulations or pressed powders that exhibit a rich microstructure with many interfaces involving crystalline grains of the energetic constituent. Indeed, the cumulative surface area of the pores, whose diameters typically range from 0.1 to 100 μm and collectively comprise <2% of the formulated HE volume fraction, is vastly outweighed by the cumulative area of microstructural interfaces with HE grains.^8–13^ Although pore collapse is the primary hot-spot ignition mechanism under strong shock loading, additional hot spots formed at interfaces could play significant, yet poorly quantified roles in HE initiation, whether directly as another ignition source or indirectly by merely increasing the background temperature (and therefore reaction rates) as a field of reactive hot spots spreads through the material.

Computer simulations have played an important role in developing the conceptual physics of HE initiation through hot spots. The earliest simulations of hot spot formation date to the 1960s and employed continuum fluid mechanics equations (i.e., the hydrodynamic assumption) on a computational mesh.^14^ Modern continuum-based “grain-scale” or "mesoscale" material models that can explicitly resolve hot spot formation must incorporate many coupled physical effects. These effects include elastic and inelastic crystal mechanics, phase transitions, momentum and energy transport, and chemical reactions, all treated at varying degrees of physical approximation (see, for example, refs 15–20 ). The primary utility of these continuum grain scale models is that they can access computational domains approaching the centimeter scale with modern computers. This is currently the only practical means to perform direct numerical simulations of hot spot formation using realistic microstructures^21,22^ (e.g., from SEM images) or to directly bridge with experimental measurements of the hot spot temperature field.^23^ A shortcoming of continuum material models is that they can be prone to model-form errors^18,19^ and thus require judicious assessments of the relevant physical processes at play, including couplings among those processes.

All-atom modeling of HEs using molecular dynamics (MD) simulations is currently limited to single-micron-scale computational domains with modern computers.^24,25^ However, MD can be applied to reductionist initial-boundary-value-problem geometries with very few physical assumptions regarding material mechanics, phase transitions, transport, and chemistry. For this reason, MD simulations have proven indispensable for identifying important physical effects in hot spot formation via pore collapse, such as revealing anisotropy in the pore-collapse process,^18^ size scaling trends,^25,26^ mechanochemical acceleration of reactions,^27–29^ molecular jetting,^30^ Fourier-like thermal dissipation,^31^ and the complicated interplay between crystal strength and phase transitions.^19^ A limited but growing body of MD studies on HE interfaces indicate strong potential for unusual and unanticipated physical effects,^32–34^ including ones that can influence HE chemical reactions.^35,36^

The generalized crystal-cutting method^37^ (GCCM) is an MD simulation construction tool that has greatly simplified the preparation of initial-boundary value problems involving low-symmetry molecular crystals. The GCCM uses carefully articulated searches over a large lattice space to identify commensurate ways to inscribe oriented crystals and crystal–crystal interfaces within 3D periodic simulation cells. It has enabled studies of shock propagation along arbitrary crystallographic directions,^38,39^ anisotropic thermal transport,^40^ and surface and interface energy calculations,^34,41^ and can improve computational efficiency by identifying equivalent orthorhombic representations of low-symmetry crystals.^37^

Here we extend the GCCM by deriving expressions to characterize the tilt and twist of crystal–crystal interfaces. We apply our extensions of the GCCM to prepare a simulation cell containing an idealized grain boundary (GB) in the triclinic molecular HE TATB (1,3,5-triamino-2,4,6-trinitrobenzene) and use this cell to predict the characteristics of shock-induced hot spot formation at the GB interface. As a triclinic crystal,^42^ TATB represents a fully general application case for the GCCM. More importantly, TATB is a layered molecular crystal^42^ that exhibits what is arguably the most strongly anisotropic set of material properties among any HE in common use, including its thermal conductivity,^43–45^ thermal expansion coefficients,^46,47^ surface energies,^41,48^ elastic compliance,^44,49,50^ inelastic deformation modes,^38,39,51–53^ and an anisotropic apparent Hugoniot elastic limit (HEL).^39^ This anisotropy is expected to exaggerate effects at GBs in TATB as compared to GBs in less anisotropic materials. Our simulations reveal that hot spot formation at GBs in TATB can depend strongly on the shock propagation direction. We trace this interfacial shock anisotropy to an acceleration of the kinetics for a shock-induced volume-reducing change in state when the shock wave crosses the GB, which leads to a short-lived transient single wave that increases dissipation until it can split into a steady two-wave structure. Henceforth, we will distinguish the intrinsic anisotropy of the TATB crystal structure and its fundamental physical properties from the extrinsic anisotropy of the GB response to shock, which depends on both the specific GB studied and its orientation in the shock frame.

## 2 Methods

### 2.1 GCCM Extensions

The GCCM^37^ employs a mathematical formalism and a search-with-constraints algorithm to identify ways to inscribe an oriented single crystal in a 3D-periodic simulation cell while preserving the crystal’s translational symmetry. Both the formalism and search algorithm are readily generalized to the problem of orienting two crystal grains to form a 2D-infinite crystal–crystal interface within an overall commensurate 3D-periodic simulation cell. This latter capability was demonstrated in ref 37 for constructing a variety of interfaces within and between hexagonal, monoclinic, and triclinic crystals. As discussed below, the user implicitly specifies the GB tilt angle τ_tilt_ within the GCCM mathematical formalism, but the subset of possible twist angles τ_twist_ found for a given τ_tilt_ is determined by the unit-cell geometries of the constituent crystals and the size of the GCCM search domain. In this subsection, following a brief summary of the geometric constraints required to construct GB interfaces using the GCCM, we derive general expressions for the systematic characterization (and possible constraint) of τ_tilt_ and τ_twist_ for arbitrary GBs. Additional details regarding the GCCM search algorithms, a method to populate the simulation cell with atoms/molecules, and all GCCM source codes can be found in ref 37.

To construct an interface using the GCCM, two cut crystals described by arbitrary Bravais lattices are specified by three cell edge vectors each, namely (**x**_1_, **x**_2_, **x**_3_) for crystal 1 and (**x**_1_^′^, **x**_2_^′^, **x**_3_^′^) for crystal 2. The vectors **x**_*j*_ and **x**_*j*_^′^ are required to be lattice vectors, so

$$\begin{array}{rl} x_{j} & =m_{j}a+n_{j}b+p_{j}c,\\ {x^{\prime }}_{j} & =m_{j}^{\prime }a^{\prime }+n_{j}^{\prime }b^{\prime }+p_{j}^{\prime }c^{\prime } \end{array}$$ 1

where **a**, **b**, and **c** are the unit cell lattice vectors for crystal 1, **a**′, **b**′, and **c**′ are the unit cell lattice vectors for crystal 2, and *m*_*j*_, *n*_*j*_, *p*_*j*_, *m*_*j*_^′^, *n*_*j*_^′^, and *p*_*j*_^′^ are integers to be determined later through application of geometric constraints. It is assumed that both sets of cell vectors form a right-handed basis; that is, **x**_1_ × **x**_2_ lies in the same half-space as **x**_3_ for crystal 1, and similarly for crystal 2. The angles between the cell vectors are defined as θ ∠**x**_1_ **x**_2_, ϕ ∠ **x**_1_ **x**_3_, and ψ ∠ **x**_2_ **x**_3_, and similarly for θ′, ϕ′, and ψ′. Orientations for the two crystals are specified by the vectors **S** and **S**′, which are either parallel or antiparallel to the normal vectors for the crystal faces forming the GB of interest. Specific integers *m*_1_,..., *p*_3_^′^ for a particular commensurate solution are found by looping over a user-specified domain (e.g., – 15 ≤ *m*, *n*, *p* ≤ 15) and checking whether the constraints are satisfied within a given user-specified tolerance.

Candidates for a complete solution (**x**_1_, **x**_2_, **x**_3_)|(**x**_1_^′^, **x**_2_^′^, **x**_3_^′^) are first identified by finding vector pairs (**x**_1_, **x**_2_) and (**x**_1_^′^, **x**_2_^′^) such that the crystal faces specified by **S** and **S**′ are aligned with each other and with a face of the simulation cell. That is, we require that

$$\begin{array}{rl} x_{1}\times x_{2} & ∥S,\\ x_{1}^{\prime }\times x_{2}^{\prime } & ∥S^{\prime } \end{array}$$ 2

The transverse dimensions of the two cut crystals are guaranteed to be commensurate if

$$\begin{array}{rl} \left|x_{1}\right| & =|x_{1}^{\prime }|,\\ \left|x_{2}\right| & =|x_{2}^{\prime }|,\\ \theta & =\theta^{\prime } \end{array}$$ 3

where |**x**| is the length of vector **x**. All unique combinations of the independently identified pairs (**x**_1_, **x**_2_) and (**x**_1_^′^, **x**_2_^′^) are compared and those satisfying eq 3 are kept as solutions (**x**_1_, **x**_2_)|(**x**_1_^′^, **x**_2_^′^) for the transverse dimensions. The longitudinal dimension is set by identifying those **x**_3_ and **x**_3_^′^ that satisfy

$$\begin{array}{c} \phi =\phi^{\prime },\\ \psi =\psi^{\prime } \end{array}$$ 4

It is not necessary to require |**x**_3_| = |**x**_3_^′^|. Those **x**_1_,..., **x**_3_^′^ that simultaneously satisfy eqs 2–4 form a solution (**x**_1_, **x**_2_, **x**_3_)|(**x**_1_^′^, **x**_2_^′^, **x**_3_^′^).

The two crystal cells (**x**_1_, **x**_2_, **x**_3_) and (**x**_1_^′^, **x**_2_^′^, **x**_3_^′^) are cut from the bulk and rigid-body rotations specified by the matrices $\underline{R}$ and ${\underline{R}}^{\prime }$ are applied to the respective crystal cells so that **x**_1_, **x**_2_, and **x**_3_ are aligned with their primed counterparts in some convenient lab coordinate frame. After an appropriate translation of crystal 2 with respect to crystal 1 (usually by **x**_3_), the two crystals are then inscribed in a single 3D periodic simulation cell.

Consider the schematic in Figure 1a, which shows a single crystal prior to being cut and the pieces rotated to form an interface. The unsigned tilt of crystal 1 with respect to crystal 2 can be obtained from

$$\hat{S}\cdot {\hat{S}}^{\prime }=\cos(\tau_{\mathrm{tilt}})$$ 5

where **Ŝ** and **Ŝ**′ are unit vectors. The twist angle τ_twist_ can be obtained by comparing the transformations of a vector **T** that is invariant under tilt. For interfaces with τ_tilt_ ≠ 0° or 180°, the invariant vector lies along the tilt axis and can be computed as

$$T=\hat{S}\times {\hat{S}}^{\prime }$$ 6

For the case τ_tilt_ = 0° or 180°, **T** can be any vector in the plane with normal **S**. An interface with τ_twist_ = 0° can be formed by making the prescribed cut in Figure 1a and rotating one of the crystals by τ_tilt_ about **T**, yielding Figure 1b. Twisting one of the crystals about vector **S** by τ_twist_ results in the interface with a twist shown in Figure 1c. The twist can be calculated by comparing vectors

$$\begin{array}{c} T_{1}=\underline{R}T,\\ T_{2}={\underline{R}}^{\prime }T \end{array}$$ 7

where $\underline{R}$ and ${\underline{R}}^{\prime }$ are the rotation matrices applied to crystals 1 and 2 in the original GCCM formulation.

**Figure 1:**
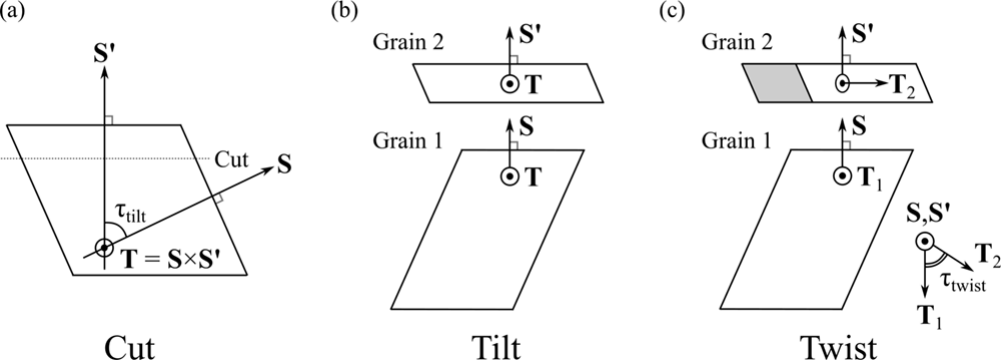
Schematics for the preparation of a crystal–crystal interface and the relation of the tilt and twist angles to transformations of vectors defined in the GCCM framework. The construction procedure involves: (a) cutting a crystal by specification of orientation vectors **S** and **S**′ which imply a tilt axis **T**; (b) rotation of crystal grain 2 by an angle τ_tilt_ about **T**; and (c) rotation of grain 2 by an angle τ_twist_ about **S**.

Two possible twists are shown in Figure 2. In the first case with **T**_1_ and **T**_2_, the twist can be directly obtained from **T̂**_1_ ·**T̂**_2_ = cos­(τ_twist_). However, in the second case with **T**_1_ and **T**_2_^′^, that metric incorrectly yields a twist angle of +(360° – τ′_twist_) when the correct twist is actually τ′_twist_ or equivalently –(360° – τ′_twist_). The correct τ_twist_ can be obtained from the conditional statement

$$\tau_{\mathrm{twist}}=\{\begin{array}{ll} +\cos^{-1}({\hat{T}}_{1}\cdot {\hat{T}}_{2}) & \mathrm{if}\;S\cdot (T_{1}\times T_{2})\geq 0,\\ -\cos^{-1}({\hat{T}}_{1}\cdot {\hat{T}}_{2}) & \mathrm{if}\;S\cdot (T_{1}\times T_{2})<0 \end{array}\;$$ 8

Note that the above definition also correctly captures both τ_twist_ = 0° and τ_twist_ = 180° = −180°. Although τ_tilt_ and τ_twist_ are most clearly understood in the context of homophase interfaces (i.e., GBs), eqs 5–8 can also be used to characterize the configuration of a heterophase interface between two dissimilar crystals.

**Figure 2:**
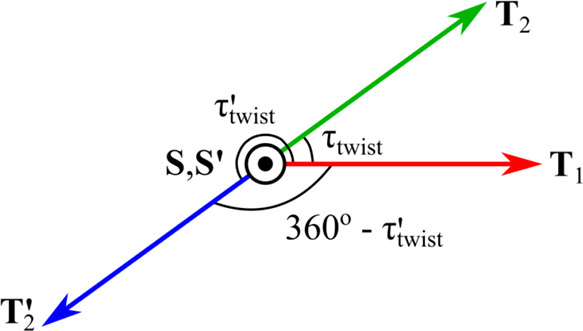
Relationship between τ_twist_ and τ′_twist_ for two possible twist axes **T**_2_ and **T**_2_^′^ attached to crystal 2. The twist angle τ_twist_ is defined via counterclockwise rotation relative to **T**_1_, so it can take on values larger than 180°.

Given a solution (**x**_1_, **x**_2_, **x**_3_)|(**x**_1_^′^, **x**_2_^′^, **x**_3_^′^) with a particular tilt and twist, such as the one shown in Figure 3, there will be three other equivalent solutions found by the GCCM. The rotations $\underline{R}$ and ${\underline{R}}^{\prime }$ were applied to (**x**_1_, **x**_2_, **x**_3_) and (**x**_1_^′^, **x**_2_^′^, **x**_3_^′^) so that in a Cartesian basis **x**_1_ = **x**_1_^′^ and **x**_2_ = **x**_2_^′^, and the primes can be dropped. It can clearly be seen that if (**x**_1_, **x**_2_) is a solution for the transverse dimensions, then there are three other right-handed solutions. These are (**x**_2_, −**x**_1_), (−**x**_1_, −**x**_2_), and (−**x**_2_, **x**_1_), and each is paired with the same **x**_3_ and **x**_3_^′^ vectors as the original (**x**_1_, **x**_2_) solution. Because each solution is cut from two crystals with identical alignment, all four solutions have the same τ_twist_. These four solutions are also equivalent from a computational efficiency standpoint as the cell angles θ and θ* are supplementary.

**Figure 3:**
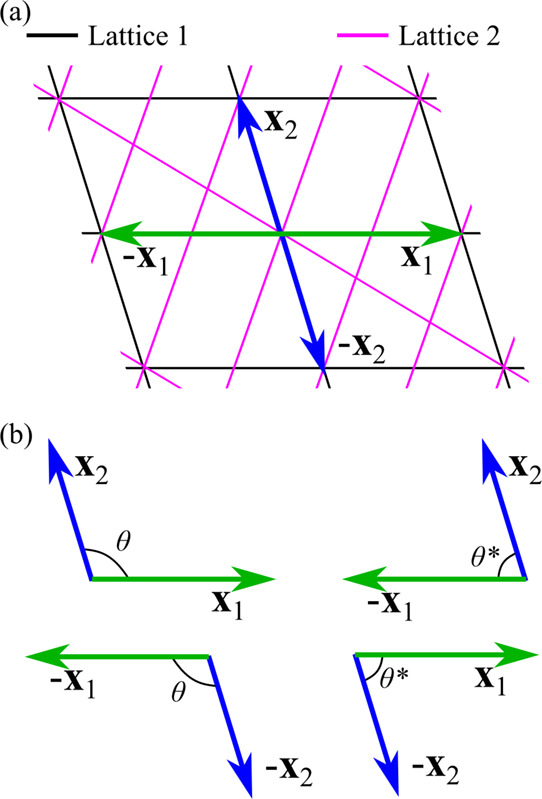
GCCM finds solutions for commensurate crystal–crystal interfaces in sets of four. (a) An example solution for a commensurate interface between two crystal lattices with specific vectors **x**_1_ and **x**_2_ for crystal 1 that were aligned with vectors **x**_1_^′^ and **x**_2_^′^ for crystal 2. (b) Four equivalent choices for **x**_1_ and **x**_2_ that form a right-handed basis.

Overall, there are five degrees of freedom (DOFs) that must be specified for idealized molecularly smooth interfaces constructed using the GCCM. The first and most significant is the tilt angle τ_tilt_, as this implicitly specifies the crystal facets that are placed in contact to form an interface. For fixed τ_tilt_, there remains four additional DOFs that must be specified. One is the twist angle τ_twist_ defined above. The next two are translations of one crystal with respect to the other in the plane of the interface. These can be visualized using the schematic in Figure 3a, wherein Lattice 2 is translated in the plane of the page while holding Lattice 1 fixed. The fifth and final DOF is the separation distance between the two crystals normal to the interface plane. All five DOF can impact the interface energetic stability and stress state, but the magnitude of this effect is likely to be system dependent and most sensitive to τ_tilt_ and the crystal separation distance. In the original GCCM report,^37^ we quantified the interfacial excess energy and stress of GBs in a molecular crystal for multiple interfaces with different τ_tilt_ (see Figure 6 in that report). These calculations showed that different τ_tilt_ resulted in GB-to-GB variations in the energy state that differed by ≈0.1 kcal/mol/atom, which is much smaller than *k*_B_ *T* at room temperature, and the excess interfacial stress varied by ≈0.1 GPa. Based on these weak dependencies for molecular crystals, we do not explore variations in these DOF here and focus instead on a single representative case. However, we emphasize that the GCCM provides a direct means to extensively explore this configurational phase space for generic crystal systems and that these DOF could play a significant role in determining interfacial properties, especially in cases involving transport (e.g., energy, charge) across an interface.

### 2.2 MD Simulation Details

All MD simulations were performed using the LAMMPS code^54^ in conjunction with an extensively validated^55^ variant^43,56^ of the all-atom, nonreactive TATB force field (FF) developed by Bedrov et al.^49^ Implementation details and example LAMMPS input files can be found in ref 55.

The anisotropic shock response of a TATB crystal with a GB was investigated using a triclinic 3D periodic simulation cell containing an idealized 2D-infinite GB between the (001) and (100) crystal faces (see Figure 4). Because TATB crystal is centrosymmetric, the faces (001) and (001̅) are equivalent, as are (100) and (1̅00). Commensurate cuts for the (001) and (100) crystal grains were identified using the GCCM^37^ in conjunction with TATB FF lattice parameters for the triclinic *P*1̅ structure^42^ corresponding to (*T* = 300 K, *P* = 1 atm), following the approach described just above. A GB was formed by placing the two grains in a triclinic cell with a 5 Å gap between them, which was chosen based on the minimum identified in a rigid-body scan of the potential energy surface probing the *z* separation distance between the two grains. The (100) grain was translated precisely along **x**_3_ when placing it on top of the (001) grain without additional transverse displacements so that the two lattices matched at the interface following the schematic in Figure 3a. The maximum magnitude strain imposed on any individual cell parameter to make the two cut grains commensurate was 0.001. The tilt and twist for the GB are respectively 78.70° and −25.42°. A small vacuum region (≈8 nm) was included to prevent the formation of a second GB due to the longitudinal periodic boundary. Excluding the vacuum region, the precompressed sample length along the shock direction was 108 nm with each grain being ≈54 nm long. The transverse dimensions were 12.6 nm × 15.5 nm, yielding a system with slightly more than 2.1 million atoms (≈90,000 molecules).

**Figure 4:**
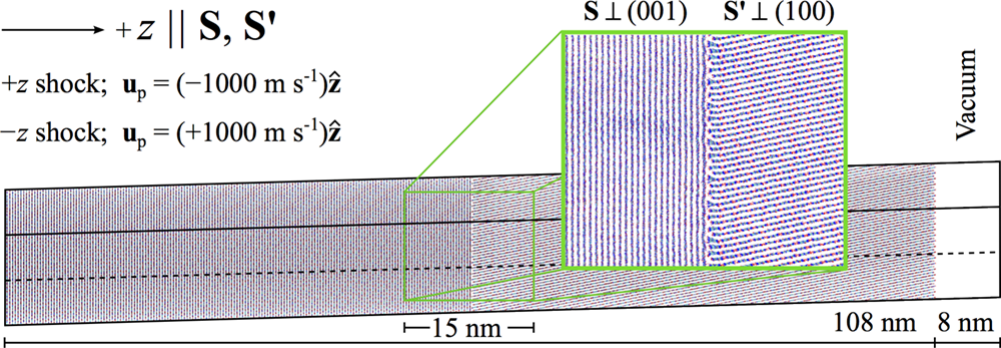
Simulation cell containing a (001)|(100) GB in crystalline TATB. The thermalized configuration of the GB is shown in the zoom-in window. Edges of the periodic cell are shown as black solid and dashed lines.

Trajectories were integrated using the velocity Verlet integrator^57^ and the equations of motion derived by Shinoda et al.^58^ Isochoric-isoenergetic (*NVE*) and isochoric-isothermal (*NVT*) trajectories were integrated using time steps of 0.10 and 0.25 fs, respectively. We used a Nosé-Hoover-style thermostat^59,60^ with a coupling parameter of 100 fs for the *NVT* trajectories. A thermal starting configuration for shock simulations was generated at *T* = 300 K through a two-step process. First, a 5 ps *NVE* trajectory was integrated during which the atomic velocities were rescaled to 300 K at 10 fs intervals, with selection of new atomic velocities from the Maxwell distribution at 100 fs intervals. This rapidly equipartitioned the potential and kinetic energy and eliminated longitudinal breathing motions along the length of the cell that result from the instantaneous introduction of a GB and exposure of free surfaces. Second, a 100 ps *NVT* trajectory was integrated, which was found to be sufficiently long for the system to reach an approximate equilibrium state characterized by a steady-fluctuating total potential energy. The initial conditions for simulations of supported shocks were set using a reverse-ballistic configuration (described below) and trajectories were integrated using *NVE* dynamics. The atomic positions, velocities, and per-atom stress tensor components were recorded at 100 fs intervals during the shock simulations.

Supported shocks were generated using a reverse-ballistic configuration^61^ and propagate along the *z* direction, which is normal to the plane of the GB. With a reverse-ballistic configuration, a large flexible sample impacts onto a thin rigid piston of the same material. Two separate shock simulations were performed with waves that propagate along the +*z* and −*z* directions and originate in the (001)- and (100)-oriented grains, respectively. For the +*z* case, we defined a two unit-cell-thick piston region at the far left surface of the (001) grain and held it rigid during the shock simulation. A net velocity **u**_p_ = −1000 m·s^–1^ was then added to the thermal velocities of the particles in the rest of the sample. This results in a supported shock wave with velocity **u**_s_ that propagates with velocity **u**_w_ = **u**_s_ + **u**_p_ in the coordinate frame fixed on the piston. A similar approach was used to generate a wave propagating along −*z*. Multiple shock-front absorbing boundary conditions^62^ (SFABCs) were applied on both ends of the system during each trajectory to capture waves transmitted through or reflected from the GB when they reach the end of the sample.

### 2.3 MD Trajectory Analysis

System properties were analyzed as functions of time *t* and position *z* along the shock direction. Spatial averages were computed for a given *t* using a moving average with a bin width of 10 Å and displacement increment of 3 Å. Molecules were assigned to the moving average bin based on their center-of-mass (COM) positions. In particular, the kinetic temperature *T*, temperature differences Δ*T* between subsets of molecular DOFs, pressure *P*, von Mises stress σ_Mises_, and components of the velocity field **v** were computed. Specific equations used to compute these properties are described below. The location of the GB was monitored by computing the average *z*-position of the molecules in the topmost planar crystal layer in the (001) grain. The uncertainty in this metric for the GB position never exceeded ≈2 Å, even under plastic deformation of that crystal layer. Trajectory snapshots were rendered using the Open Visualization Tool (OVITO).^63^

The velocity field **v** for a given spatial bin *i* was defined to be the net COM velocity of that bin **v**_cm,*i*_. The kinetic temperature for a given bin *T*_*i*_ was computed as

$$T_{i}=\frac{1}{3k_{B}N_{i}^{\mathrm{bin}}}\overset{N_{i}^{\mathrm{bin}}}{\underset{j=1}{\sum }}m_{j}{\left(u_{j}-v_{\mathrm{cm},i}\right)}^{2}$$ 9

where *k*_B_ is the Boltzmann constant, *N*_*i*_^bin^ is the number of atoms in bin *i*, and *m*_*j*_ and **u**_*j*_ are the mass and velocity vector of atom *j*. Similar expressions were used to compute the kinetic temperatures of the molecular translational and ro-vibrational DOF from the atomic and molecular COM velocities.^64^

By default, LAMMPS computes per-atom stress tensor components σ_αβ,*j*_ that include both the kinetic and virial terms in a stationary lab frame

$$\sigma_{\alpha \beta ,j}=-m_{j}u_{\alpha ,j}u_{\beta ,j}+\sigma_{\alpha \beta ,j}^{\mathrm{virial}}$$ 10

where α and β denote specific Cartesian directions and *u*_α,*j*_ and *u*_β,*j*_ are the velocity components for atom *j* along the α and β directions. Note that eq 10 yields a quantity that has units of GPa·m^3^ which must later be normalized by an appropriate volume *V*. Due to the kinetic term in the stress tensor, this definition does not yield the actual stress state for parts of a system undergoing rigid body motion, such as the uncompressed material ahead of the shock in our reverse-ballistic simulations. However, eq 10 does correctly capture the stress state of fully compressed material because the reverse-ballistic configuration leads to zero net rigid-body motion in that part of the system which is behind the advancing shock wave. Therefore, local postshock mass flow due to inelastic deformation behind the shock, which contributes to the kinetic part of the local stress, is correctly captured by our definition of the velocity field.

The stress state of the material was characterized using scalar metrics proportional to the first and second rotational invariants of the stress tensor, namely the pressure *P* = – Tr­[**σ**]/3 and von Mises stress

$$\sigma_{\mathrm{Mises}}={\left[\frac{1}{2}{\left(\sigma_{11}-\sigma_{22}\right)}^{2}+{\left(\sigma_{22}-\sigma_{33}\right)}^{2}+{\left(\sigma_{33}-\sigma_{11}\right)}^{2}+6\left(\sigma_{12}^{2}+\sigma_{23}^{2}+\sigma_{31}^{2}\right)\right]}^{1/2}$$ 11

Here Tr­[**σ**] denotes the trace of the stress tensor **σ** computed within a given bin *i* as

$$\sigma_{i}=\frac{1}{V^{\mathrm{bin}}}\overset{N_{i}^{\mathrm{bin}}}{\underset{j=1}{\sum }}\sigma_{j}$$ 12

from the per-atom stress tensors **σ**_*j*_ computed using eq 10, normalized by the constant bin volume *V* ^bin^. We note that quantities which are proportional to the number density, including the above expressions for the stress, exhibit nonphysical fluctuations which arise due to the discrete atomic nature of the system. Bin widths between 5 and 30 Å were considered and 10 Å was chosen as it yielded signals with acceptable noise without obscuring fine details. Quantities such as the temperature and velocity field do not exhibit these density-induced fluctuations as they are respectively normalized by the nonconstant particle number *N*_*i*_^bin^ and total bin mass *M*_*i*_^bin^.

The shock front position at a given time *t* was determined by monitoring the COM position of the bin with the maximum gradient of the kinetic energy along the shock direction.^64,65^ Material within the fixed regions at either end of the system was excluded from this analysis. We also excluded ≈1 nm of material behind each front when computing shock velocities for multiwave structures to avoid possible convolution of the fronts.

## 3 Results and Discussion

### 3.1 Wave Structures and Stress States

Two different shock simulations were performed using the TATB GB cell in Figure 4 in which the shock either (1) originates in the (001) grain and propagates along +*z* or (2) originates in the (100) grain and propagates along −*z*. Comparisons of the resulting pressure state are shown in Figure 5 for shocks that propagate along +*z* in panel (a) and −*z* in panel (b). Analogous plots of the von Mises stress are shown in panels (c) and (d) for the +*z* and −*z* shocks, respectively. It should be noted that the system is actually in a state of tension (≈ −1 GPa) prior to shock compression, although the net translation leads to an apparent state of compression (≈ +1 GPa) in the figure through the kinetic contribution to the stress. Similar tension states were predicted for energy-minimized GB configurations in β-HMX^37^ and stem from both the GB and the fact that both crystal grains were strained to inscribe them in a single 3D periodic simulation cell. We chose not to apply a barostat during the system equilibration as there are significant compliance differences between the grains in the transverse dimensions, which could lead to undesirable inhomogeneous lattice strains under the influence of an MD barostat that responds only to the system average stress tensor.

**Figure 5:**
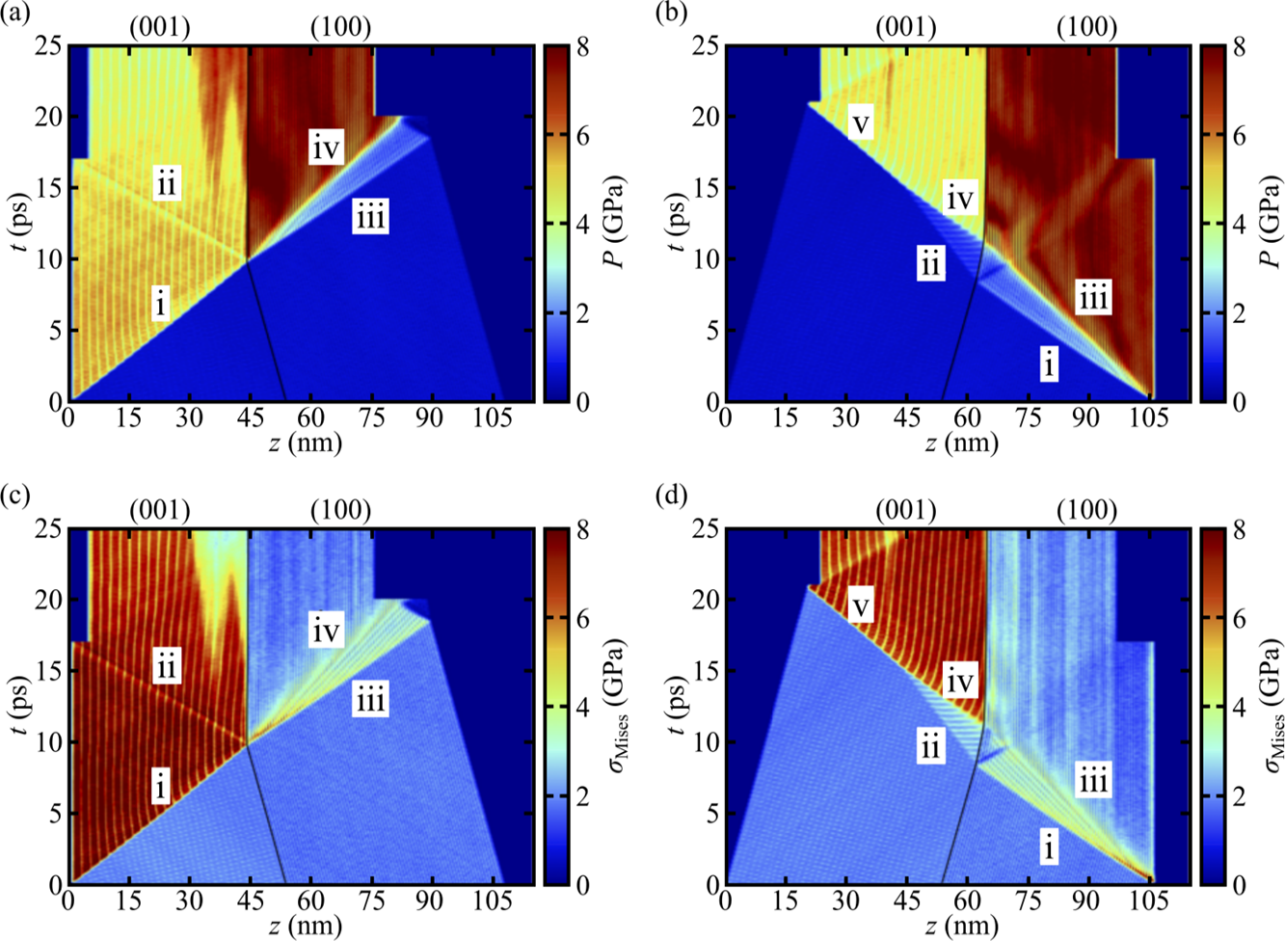
Top row: system pressure *P* as a function of time *t* and coordinate *z* along the shock direction for shocks that propagate along (a) +*z* and (b) −*z*. Bottom row: analogous plots of von Mises stress for the (c) +*z* and (d) −*z* cases, respectively. The location of the GB is shown as a solid black curve and the various wave features described in the text are indicated with Roman numerals.

We focus first on the complicated wave structures that arise due to the presence of a GB and the substantial anisotropy in the apparent HEL for the two crystal orientations. In Figure 5a, which is for the +*z* case, a single elastic wave (see i) propagates at +4550 m·s^–1^ through the (001) grain until it reaches the GB. At the GB, part of the incident wave is reflected back into the (001) grain, leading to another elastic wave (see ii) traveling at −6230 m·s^–1^ that is later captured by an SFABC applied near the piston at *t* ≈ 17 ps. The rest of wave i transmits through the GB, leading to an elastic wave (see iii) that propagates at +5040 m·s^–1^ in the (100) grain. The strength of wave iii is above the HEL for shocks normal to (100), which results in a slower secondary inelastic wave (see iv) that originates at the GB and propagates at +3760 m·s^–1^ toward the end of the system. Both waves iii and iv are captured by an SFABC that is applied to the right-hand end of the sample when it reaches maximum compression at around *t* = 17 ps.

More complicated wave structures arise when the shock propagates along the −*z* direction, as shown in Figure 5b. First, a primary elastic wave (see i) propagates at −5260 m·s^–1^ through the (100) grain until it reaches the GB. Most of this wave is transmitted, leading to an elastic wave that propagates at −2690 m·s^–1^ in the (001) grain (see ii). Wave ii is slower than wave i in part because the elastic response of TATB is significantly stiffer normal to (100) than normal to (001).^49^ Similarly, the strength of wave i is above the HEL for the (100) grain, resulting in a secondary inelastic wave (see iii) that propagates toward the GB at −3850 m·s^–1^. Both waves i and iii have small reflections at the GB that are captured by an SFABC near the piston at *t* ≈ 16 ps. Much of wave iii transmits through the GB, leading to a second elastic wave (see iv) in the (001) grain. Wave iv travels much faster (−5160 m·s^–1^) than wave ii because it propagates through material that has already been compressed. At *t* ≈ 15 ps, wave iv overtakes wave ii, resulting in a single elastic wave (see v) that propagates at −4150 m·s^–1^ until it is captured by an SFABC when the system is at maximum compression (*t* ≈ 21 ps).

Comparing Figure 5a,b shows that the postshock stress state is highly anisotropic with respect to shock propagation direction. In both the +*z* and −*z* cases, the (001) grain exhibits a pressure of ≈4 GPa whereas the (100) grain is at ≈8 GPa. Signatures for inelastic deformation of the crystal are present in plots of the von Mises stress, σ_Mises_, shown in panels (c) and (d) of Figure 5. High σ_Mises_ indicates significant local shear stresses. Comparison of panels (a) and (b) to panels (c) and (d) in Figure 5 reveals that postshock decreases in σ_Mises_ are accompanied by increases in pressure. Clearly, the (001) grain supports significantly higher shear stresses than does the (100) grain. For the +*z* case, at *t* ≈ 15 ps there are two independent inelastic deformation nucleation events in the (001) grain, with one at *z* ≈ 38 nm and the other at the GB. In the −*z* case, there is only a single nucleation event which occurs far from the GB; this is most apparent in panel (b), near the location where waves ii and iv coalesce into wave v at *t* ≈ 23 ps and *z* ≈ 40 nm. The response of the (100) grain in both shock direction cases is quite similar, namely, a lead elastic wave resulting in relatively small σ_Mises_ that quickly relieves forming a secondary inelastic wave. We note that in the (100) grain, σ_Mises_ is highest near the GB in the +*z* case and near the piston in the −*z* case. The consequences of this shear stress localization will be discussed at length in Section 3.4.

### 3.2 Initial Thermal Response

The kinetic temperatures for the +*z* and −*z* cases are shown in Figure 6 panels (a) and (b), respectively, and the corresponding differences Δ*T* between the kinetic temperatures of the molecular translational and ro-vibrational DOF are shown in panels (c) and (d), respectively. It is clearly seen that the thermal shock response is highly anisotropic, with the formation of a hot spot at the GB only in the +*z* case. This hot spot forms exclusively from mechanical work as our simulations are nonreactive and are also *NVE*. Part of the total work done is the result of adiabatic compression, but one or more inelastic deformation processes could also serve as sources of heat. The specific mechanisms that contribute to anisotropic hot spot formation and growth at the GB are discussed more extensively in Sections 3.3–3.5.

**Figure 6:**
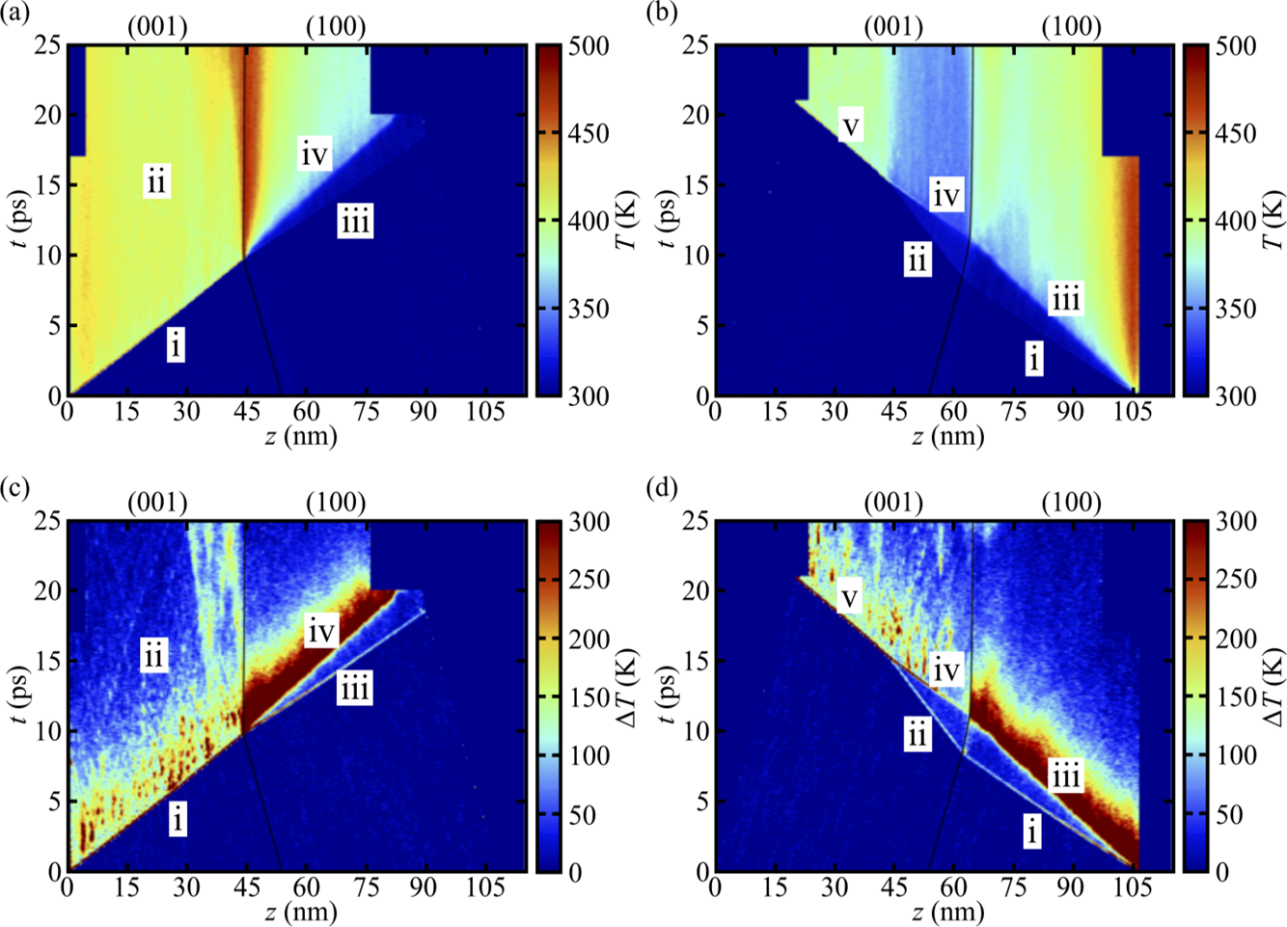
Top row: kinetic temperature *T* as a function of time *t* and coordinate *z* along the shock direction for shocks that propagate along (a) +*z* and (b) −*z*. Bottom row: analogous plots depicting the difference between the kinetic temperatures of the molecular translational and ro-vibrational degrees of freedom, Δ*T*(*z*, *t*) = *T*_trans_(*z*, *t*) – *T*_ro‑vib_(*z*, *t*), for the (c) +*z* and (d) −*z* cases, respectively. The location of the GB is shown as a solid black curve and the wave features previously identified in Figure 5 are indicated with Roman numerals.

There is considerable anisotropy in the single-crystal response to shock compression. One clear difference is that there is significant overheating in the (100) grain near the rigid piston compared to regions far from the piston, but practically no overheating near the piston in the (001) grain. Overheating at the interface between the flexible sample and rigid piston is typically seen in simulations of shocked molecular crystals and is usually attributed to a nonequilibrium effect. Perhaps the most interesting feature is that the distinct overheating at the piston in the (100) grain in the −*z* case appears very similar to the hot spot formed at the GB in the +*z* case. We will revisit these similarities in the following subsections.

The lead wave i in the (100) grain for the −*z* case results in rapid but minimal heating (by ≈20 K, barely perceptible to the eye in Figure 6b), with a persistent difference Δ*T* between the kinetic temperatures for the translational and ro-vibrational DOFs (see panel d). The temperature jump due to wave iii is greater and is primarily the result of work done to inelastically deform the crystal. The final temperature in the middle of the (100) grain is *T* ≈ 383 K. We note that there is an approximate 5 ps delay in the equilibration of the translational and ro-vibrational DOF, which contributes to a detectable delay in completing the temperature rise behind wave iii in panel b. Similar phenomena were documented in a study of crystalline nitromethane^66^ and have also been observed in the shock rise in single-crystal TATB.^39,67^ This phenomenon is apparently quite general, having been first quantified in simulations of monatomic Lennard-Jones fluids,^68^ but it is particularly marked in organic materials. Mathematical formulations for this relaxation process in simple atomic systems have recently been developed.^69^

In contrast, the lead wave i traveling through the (001) grain in the +*z* case (see Figure 6a,c) results in significant and highly efficient heating, with a temperature jump of over 100 K that occurs on a subpicosecond time scale. This temperature increase is dominated by a large and fast increase in the kinetic energy of the intramolecular DOF. However, there is also an ≈5 ps time period for equilibration between the translational and ro-vibrational DOF, which contributes to a detectable rise in the final temperature of the material. Near-instantaneous heating of intramolecular DOF is seemingly in contrast to the standard theory for localization of energy in those DOF via phonon up-pumping during shocks,^70^ which assumes that shocks couple primarily to molecular translational DOF with delayed heating of intramolecular DOF via anharmonic mode coupling. Indeed, there is some overheating of the translational DOF in the (001) grain at the primary wavefront (*T*_trans_ ≈ 800 K), but those DOF equilibrate with the remaining DOF within ≈5 ps, as can be seen in panels (c) and (d) in Figure 6. However, this relaxation process only leads to a small increase in the final temperature, due to the large number (69) of ro-vibrational DOF per TATB molecule versus only three translational DOF per molecule.

One possible explanation for this anisotropic shock heating in the single crystal is that intramolecular vibrational modes may couple more strongly to shocks that propagate normal to (001) as compared to (100). TATB is a planar molecule with 21 out-of-plane vibrational modes. The atomic displacement vectors associated with those modes are exactly parallel to *z* in the (001) grain, which could facilitate coupling to the shock. Many of these out-of-plane modes are also of low vibrational frequency that fall within the “doorway” region in the up-pumping framework.^71^ In contrast, because the normal to (100) is not parallel to the crystallographic direction [100], none of the other 45 in-plane modes have displacement vectors aligned exactly with the shock in the (100) grain.

Another phenomenon of note is apparent in Figure 6b. As discussed above, a two-wave elastic-plastic wave structure (i and iii) arises in the (100) crystal for the −*z* shock. Upon transmission into the (001) crystal, a two-wave structure (ii and iv) is initially created. However, because the (001) crystal only supports an elastic wave structure at these pressures on this time scale, the second, higher-pressure shock wave (iv) overtakes the slower first wave (ii) and coalesces into a single wave (v) at *t* ≈ 15 ps. The point that we emphasize here is that the two-wave structure (ii and iv) results in a smaller temperature rise (≈50 K) compared to a rise by ≈100 K when those two waves coalesce into a single wave (v). This will be discussed further below.

### 3.3 Deformation Mechanisms

Several inelastic deformation mechanisms are activated in the TATB GB system that are potential sources of heat. Three of these mechanisms are known to arise in TATB single crystals, namely, buckling of the molecular layers in the (100) grain, the formation of shear bands in the (001) grain, and layer sliding in the (001) grain. Activation of these mechanisms is known to depend on the loading direction relative to the crystal, the strain rate, and the background pressure state.^52,53^ For example, layer buckling primarily arises when axial loading is aligned within the plane of the molecular layers, as is the case for the (100) grain, and the critical shear stress for activation is on the order of 1–2 GPa, which is well below the shear stress induced by the shock.^52^ Shear banding arises when axial loading is oriented normal to the layers, as is the case for the (001) grain. The required loading stress for activation is greater (>4 GPa)^53^ and the kinetics for shear band nucleation can be slow relative to the shock rise time for weak shocks.^39,72^ Layer sliding can activate with sub-GPa shear stresses, but it does not lead to substantial increases in local kinetic and potential energy, and is thus not a significant heat source.^53^ Focused discussions on TATB single-crystal deformation mechanisms are given in earlier reports.^39,52,53^ An additional deformation mechanism also arises due to the GB, which involves cracking of the (001) crystal layer at the GB interface. Figure 7 shows a time sequence of snapshots taken from the +*z* shock case that illustrates the nucleation and growth of crystal defects.

**Figure 7:**
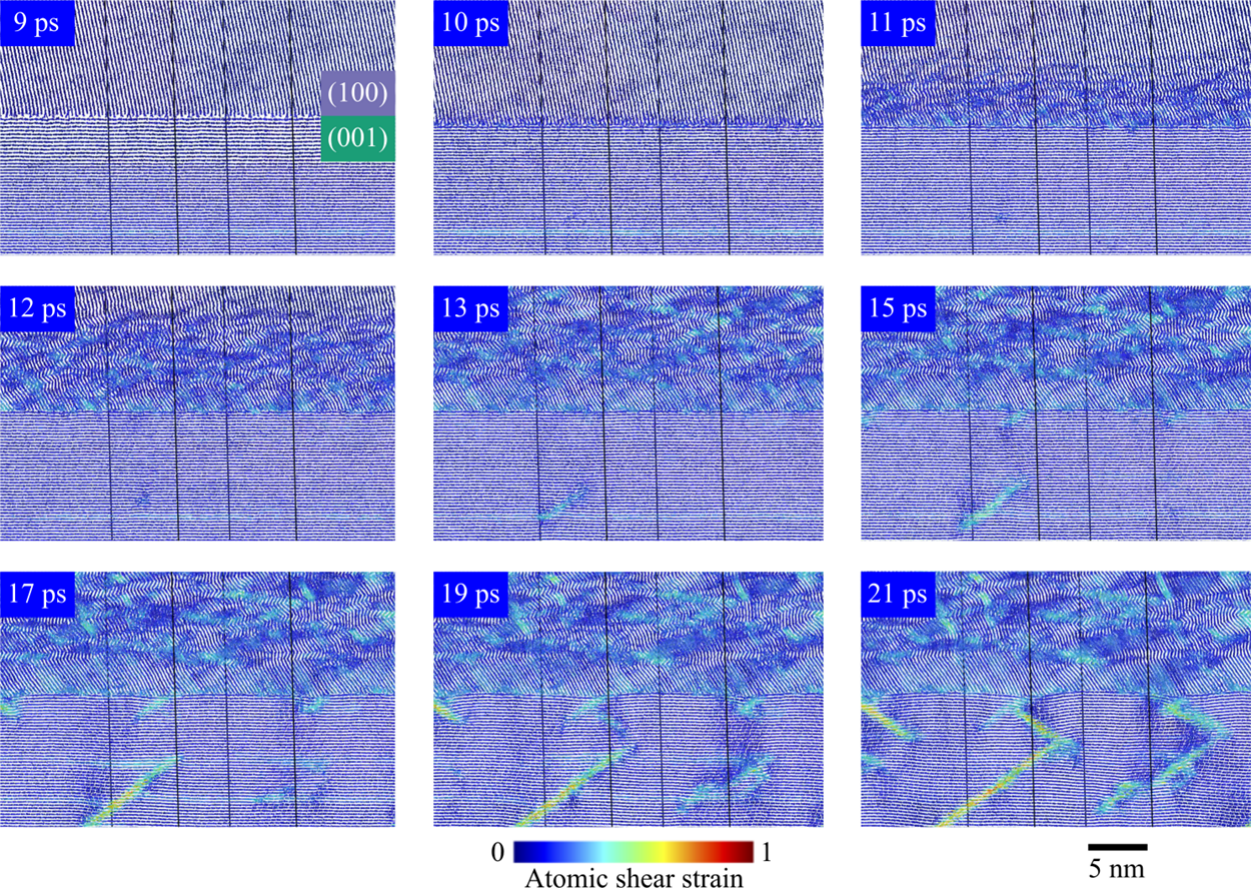
Time sequence of trajectory snapshots showing defect nucleation and growth for the +*z* shock case, starting from 1 ps prior to arrival of the shock at the GB,which occurs at physical time *t* = 10 ps. A 2 nm planar slice of the simulation cell is shown and the atoms are colored by atomic shear strain as computed using OVITO.^63^ Periodic repetition is not clearly apparent in the images, as the normal for the slicing plane does not coincide with the normal vector of a simulation cell face.

Once the +*z* shock transits the GB and enters the (100) grain, the crystal layers in the (100) grain immediately begin to buckle and eventually form an irregular corrugated structure with many subdomains. Unlike for the crystal prior to shock arrival, the layered subdomains in the shock-compressed crystal only extend a few nanometers into or out of the page and can only be clearly seen in thin slices of the simulation cell. The seemingly disordered regions correspond to multiple subdomains that are superimposed on each other in the projection. Over time, the buckling defects evolve and the individual subdomains grow in size. Similar migration and growth of the domains was observed in MD simulations of TATB crystal subject to a dynamic compressive load at a constant true strain rate.^53^ In the present case, this nanostructural evolution is driven by slip-less sliding in the plane of the GB, as is evident in maps of the velocity field shown in the Supporting Information.

Much slower shear band formation processes occur in the (001) grain. There are two distinct nucleation points, one at the GB and the other in the bulk crystal, which are evident by *t* = 15 ps. Nucleation of the shear band that forms in the bulk crystal can be detected as early as *t* = 12 ps and both nucleation sites are evident by *t* = 15 ps. The shear band in the bulk region propagates at an angle of 56.5° with respect to the shock direction, which is similar to the shear bands formed in other MD simulations of shocked oriented TATB crystal.^38,39,72^ Nucleation and growth of the shear bands is clearly evident from the significant excitation of molecular translational DOF seen in Figure 6c, for *z* ≈ 30–45 nm commencing at *t* ≈ 15 ps. A corresponding relief of the von Mises stress and increase in volumetric stress can be seen in panels (a) and (c) of Figure 5. A band-like region in the (001) grain encompasses an entire crystal layer in the *x*–*y* plane and exhibits moderate (≈0.4) shear strain. This region corresponds to layer sliding, which is not as effective as shear banding for generating heat.^53^

The primary deformation mode for the (100) grain is buckling of the molecular layers, which is at least partially reversible. A simulation of shock unloading was performed in which the −*z* shock trajectory was integrated beyond maximum compression without application of SFABCs. Snapshots from this trajectory are shown in Figure 8. As seen in the bottom snapshot, there is almost complete recovery of the original layered crystal structure. This reversibility is consistent with the interpretation that layer buckling occurs due to elastic instability of the TATB crystal lattice owing to the large difference in elastic compliance along directions within and normal to the crystal layers.^73^

**Figure 8:**
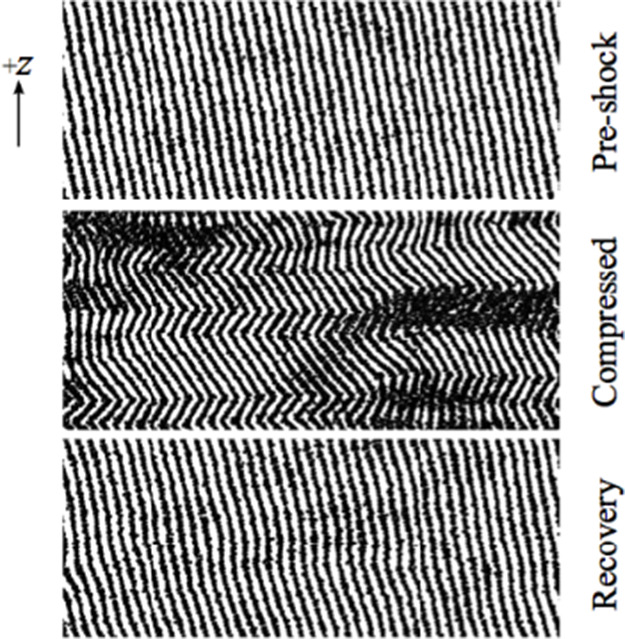
Buckling of the crystal layers in the (100) grain and recovery upon unloading. A 2 nm planar slice of the simulation cell is shown to reveal structure in the nanoscale subdomains.

One difference between the +*z* and −*z* cases is the response of the crystal layers in the (001) grain near the GB. Figure 9 shows a comparison of the topmost crystal layer in the (001) grain for the two shock directions. This crystal layer is significantly damaged in the +*z* case, and exhibits patterns reminiscent of a shattered glass pane with substantial molecular rotational disorder. The layer is also warped out-of-plane along the shock direction. In contrast, the −*z* case shows substantially less cracking and out-of-plane deformation. It should also be noted that the deformation in the +*z* case occurs almost instantaneously when the shock passes through the GB, whereas the cracking evolves more slowly (over ≈10 ps) in the −*z* case. The qualitative difference in damage of the topmost (001) crystal layer is correlated with the difference in local heat generation at the GB. However, this alone cannot simultaneously explain the formation of hot spots in the (100) grain at the GB in the +*z* and at the piston in the −*z* case.

**Figure 9:**
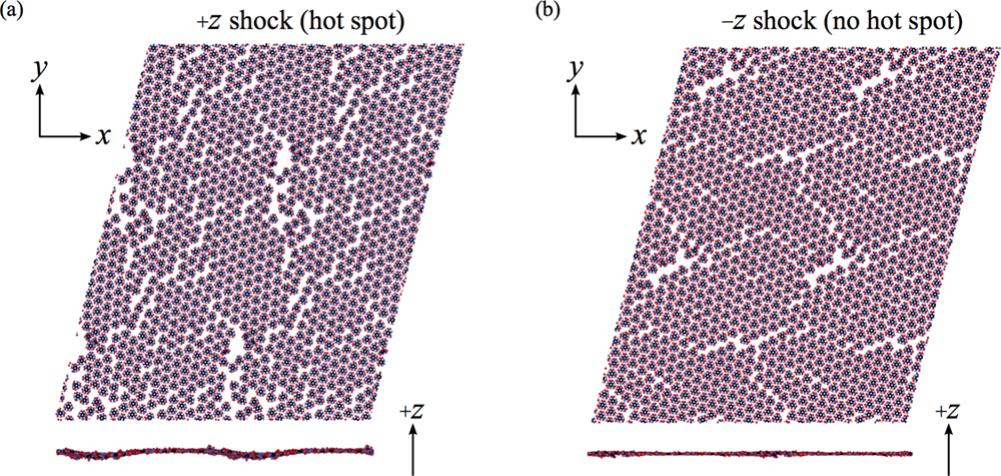
Comparison of the topmost layer of the (001) grain at the GB for the (a) +*z* and (b) −*z* cases at time *t* = 25 ps. The top and side views in each panel show a 2 × 2 repetition of the simulation cell to emphasize the deformation structure.

### 3.4 Explanation for Interfacial Hot Spot Anisotropy

The above observations and analyses indicate that hot spots form near interfaces in the (100) crystal grain, but only when the (100) grain is on the receiving end of a passing shock wave. That is, a hot spot forms in the (100) grain at the GB in the +*z* case when the shock passes from the (001) grain to the (100) grain, but not vice versa. Similarly, a hot spot forms at the interface with the piston in the −*z* case, but no hot spot forms in the (001) grain at the piston in the +*z* case. Interplay of multiple shocks and with the GB also leads to an abrupt change in homogeneous heating characteristics in the (001) grain for the −*z* case far from the GB interface. Here we articulate physical explanations for this observed interfacial shock anisotropy.

There are several consistent features for both the +*z* and −*z* case hot spots near their respective GB and piston interfaces. (1) The hot spot region is on the order of 5 nm thick and is initially contained entirely within the (100) grain. (2) While a two-wave shock profile eventually develops in the (100) grain, there is little-to-no separation between these waves in the hot spot region. (3) The von Mises stress relaxes very quickly within O­(1 ps) within the hot spot region, coinciding with analogously prompt buckling deformations. The time lag for shear stress relaxation increases as a two-wave feature develops. (4) The peak von Mises stress is greatest in the (100) grain within the hot spot region. For example, in the +*z* case, the lead wave iii results in σ_Mises_ ≈ 6 GPa near the GB whereas σ_Mises_ ≈ 4 GPa further away. Similarly, in the −*z* case, wave i leads to σ_Mises_ ≈ 8 GPa near the piston and σ_Mises_ ≈ 4 GPa further away. (5) The time scale to reach a steady state in the buckled (100) grain is at least 5 ps, as evidenced by the disequilibrium between the translational and ro-vibrational temperatures measured by Δ*T*. (6) The final pressure state is the same across the entire (100) grain. From these observations, we conclude that hot spots develop in the (100) grain precisely in regions where the shock wave induces prompt buckling deformations and does not cleanly split into a steady two-wave feature.

Figure 10 shows schematics of the shock Hugoniots for possible one- and two-wave structures in the pressure–volume (*PV*) plane, and depicts graphically how those wave structures will affect the resulting temperatures achieved in the two scenarios. We discuss each in turn.

**Figure 10:**
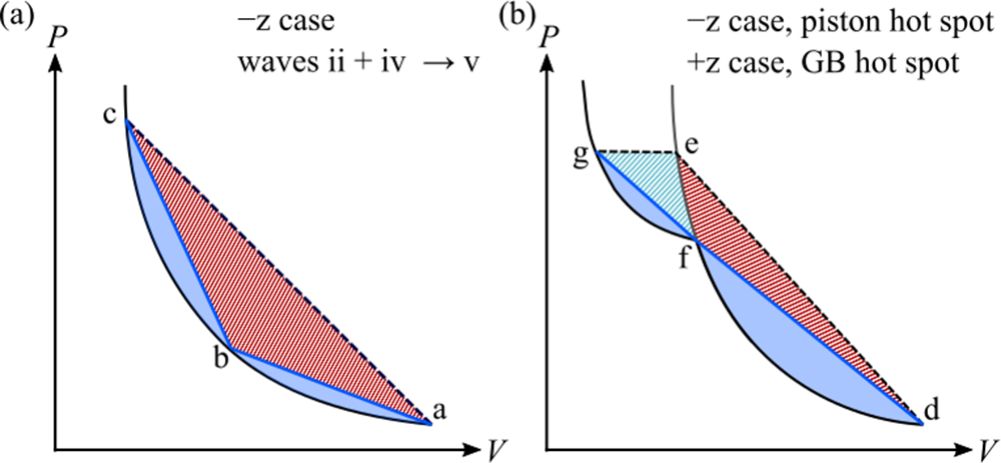
Schematic *P*–*V* Hugoniot diagrams for possible one- and two-wave structures in materials. (a) Difference between a double-shock (a→b followed by b→c) and single shock (a→c) to reach the same final shock pressure *P*_c_. (b) Possible paths for an elastic-plastic wave structure to reach the same final shock pressure *P*_g_. In both cases, the cross-hatched areas represent the extra energy that would be deposited in a single-shock process as compared to the double-shock one, for which the deposited energy is indicated by the solid shaded regions.

The case in Figure 10a corresponds to the wave structures in the (001) grain for the −*z* case, which produced the temperature profile in Figure 6b. Here, a two-wave elastic-plastic structure first develops in the (100) grain and that structure is subsequently transmitted into the (001) grain. As described above, the shock strength is not sufficient to achieve prompt plastic deformations in the (001) grain, which leads to a simple two-wave structure. Because the second shock (b→c) has a higher velocity than the first shock (a→b), the former eventually overtakes the latter and consolidates into the single a→c shock. The material that is singly shocked to the final state c has a much higher temperature than the doubly shocked material shocked to the same final *PV* compression state; compare the temperature behind wave v in Figure 6b to those behind waves ii and iv. We note that the wave velocity of the final wave (proportional to slope of the line ac) is intermediate between those of the other waves (proportional, respectively, to the slopes of lines ab and bc). Rigorously, the second wave should follow a second, distinct Hugoniot that originates at point b and then tracks slightly to the right of the drawn Hugoniot because the preshocked material is hotter and more difficult to compress as compared to unshocked material.^74^ This should be a small correction, however, and we ignore it here as do many others elsewhere.

The case in Figure 10b applies when the shock wave first enters the (100) grain and produces a hot spot either in the (100) grain at the GB interface (in the +*z* case) or in the (100) grain at the interface with the piston (in the −*z* case). In both cases, the shock wave is of the sufficient strength *P*_g_ that an elastic-plastic two-wave feature should develop. (More precisely, the second wave corresponds to a volume-decreasing transition to a state where the TATB crystal layers buckle due to an elastic instability.^73^) However, there is a time/spatial delay in the development of the two-wave structure on the order of 2 ps/5 nm, which gives rise to an overdriven elastic wave. Such phenomena have previously been observed in metals and models for this have been developed.^75–77^

Generally, the elastic wave does not need to be completely overdriven to the maximum pressure, *P* = *P*_g_. Rather, the initial shock state could be anywhere on the initial Hugoniot locus between points f and e, from which the trailing plastic wave takes the material to point g in the *PV* space. Such cases can be found in the literature on shocked molecular crystals as well as our own simulations. For example, a recent MD study on shocked crystalline HE RDX showed the evolution to steady-state of an elastic-plastic wave where the initial state at the interface appeared to be an overdriven elastic state.^78^ That same study examined the sensitivity of plasticity nucleation and development in RDX to whether the piston is modeled as a smooth reflective wall versus an explicit stationary slab of rigid molecules comprising an initially structurally commensurate piston/sample interface, and determined that the latter approach delayed the onset of plasticity compared to the former. Experimentally, somewhat overdriven elastic waves are often observed, as shown with HMX,^79^ and the models cited above presume that defect concentrations would assist with the nucleation of plasticity.

Our simulations employ explicit molecular pistons. Therefore, the initial shock coming the (100) grain from the right (i.e., the −*z* case) should provide the maximum delay for the onset of plasticity, because the (100) grain impacts onto an initially structurally commensurate (100)-oriented piston. In comparison, for the wave coming into the (100) grain from the shocked (001) grain (i.e., the +*z* case), the GB structure is somewhat irregular, which could possibly help nucleate plasticity. However, the temperature rise for both situations is ≈150 K and occurs over a distance of ≈5 nm. We infer that both sides are maximally overdriven with a large amount of excess heat being deposited near the interfaces. What is perplexing is that the von Mises stress in both cases is very quickly relaxed (<1 ps), as can be seen in Figure 5 panels (c) and (d); and with that relaxation, the two-wave pressure structure is also quickly established. As such, this phenomenon does not appear to completely account for the broader region of material at elevated temperature.

Another aspect for consideration is the effective finite shock rise time in organic materials. Although the pressure rise is fairly prompt, in terms of temperature, there is a finite delay time before the shocked material comes to an equilibrium partitioning of kinetic energy among the various lattice and molecular vibrational phonon modes. This is often referred to as vibrational up-pumping and was first well-documented in simulations of nitromethane^66^ and has subsequently been documented in TATB.^39,67^ As also shown here, this process requires 3–5 ps in TATB. Recent mode-selective pump–probe spectroscopy measurements have been performed on a variety of related organic molecular explosives, and these all show a broad spectrum of vibrational relaxation time scales.^80–82^ Those measurements showed that most mode excitations attenuate significantly within 5–10 ps, in very good agreement to what we observe here for TATB under the classical approximation. This is an excellent confirmation of the presence and significance of this phenomenon. The time delay in the equilibration can be thought of in terms of a viscosity factor for the redistribution of energy.^68,69^

In those terms, the resulting physical response of the material in the MD simulations is analogous to what is observed in continuum hydrocode simulations that use an artificial viscosity, which produces a finite shock rise time. In such simulations, a phenomenon generally described as an “entropy error” results when a shock encounters an interface between two materials of different impedance.^83,84^ When a reflective shock is sent back into the initial material, the physical expectation is a relatively small temperature rise corresponding to the two-step shock process shown in Figure 10a. However, when this process occurs with a shock wave having finite width, there will exist a zone of similar width near the interface where the material only “perceives” a single shock that takes it to the final higher pressure for the reflection. Consequently, that zone will then be at a much higher temperature than otherwise expected. For continuum-level modeling, this has spurred development of algorithms that do not utilize artificial viscosity in order to avoid this error.

However, organic materials have a very definite real viscosity, both in terms of the conventional sense of molecular momentum exchange and also in the current sense of energy equilibration encountered here. Consequently, there is a very real “error” in the deposition of excess energy around interfaces, particularly in the lower impedance material. We emphasize that the energy relaxation time of 3–5 ps corresponds to the hot zone of ≈5–10 nm, and that this does not explicitly require an elastic-plastic wave structure. This phenomena likely contributes to the excess energy observed when a shocked crystal impacts a quiescent crystal, when shocks traverse phase boundaries, and when reflected/rarefaction waves closely coincide with a primary wave.^32,33,36^

Related phenomena were first characterized for shocks in Ar gas where it was noted that it takes a finite (and calculable) amount of time for a Boltzmann distribution of translational velocities to be re-established after the passage of a shock wave.^85^ A summary of various solutions for simple materials under different boundary conditions has been summarized, including calculated profiles of the resulting temperature discrepancies.^86^ For the current organic solids, there are multiple time scales associated with the molecular translational/phonon modes (10–100 cm^–1^) and the range of vibrational frequencies (100–3000 cm^–1^) which renders a precise analysis difficult. We note that for MD-informed grain-scale continuum simulations, one should directly employ such a viscosity term to prevent over-resolution and represent the physics more accurately.^87^ Similarly, viscosity terms for this equilibration should be added to particle-based coarse-grained simulations.^88^

The small thickness of these hot spots (only a few nm) would require a very high temperature (>1500 K) for them to become self-sustaining by common estimates for chemical kinetics of secondary explosives.^3^ Though the hot spot temperature in our classical simulations is only ≈500 K, this would be somewhat higher if the molecular specific heat were treated in a quantum mechanical manner. One estimate for the “true” hot spot temperature based on an Einstein-oscillator equation of state^67^ (EOS) for the specific heat of TATB is *T* ≈ 600 K. Experimental one-dimensional time-to-explosion experiments indicate that chemical reaction time scales are on the order of 10s of minutes at this temperature, albeit for lower pressures states than realized here.^89^ The shock pressure is also on the cusp of the experimental threshold for detonation initiation.^72,90^ Thus, while there is a possibility for chemistry at the GB, such reactions are almost certainly many orders of magnitude slower than the subnanosecond time scale of our MD simulations. These zones of elevated temperature would enhance the rates of deflagration from fronts that had been initiated elsewhere.^24^

### 3.5 Hot Spot Evolution

The hot spot identified in Figure 6a for the +*z* case initiates at the GB in the (100) grain and grows into the (001) grain. To examine the evolution of this hot spot, SFABSs were applied to the +*z* shock simulation and the trajectory was integrated to *t* = 50 ps. Extended time histories of the stress and temperature state are shown in Figure 11.

**Figure 11:**
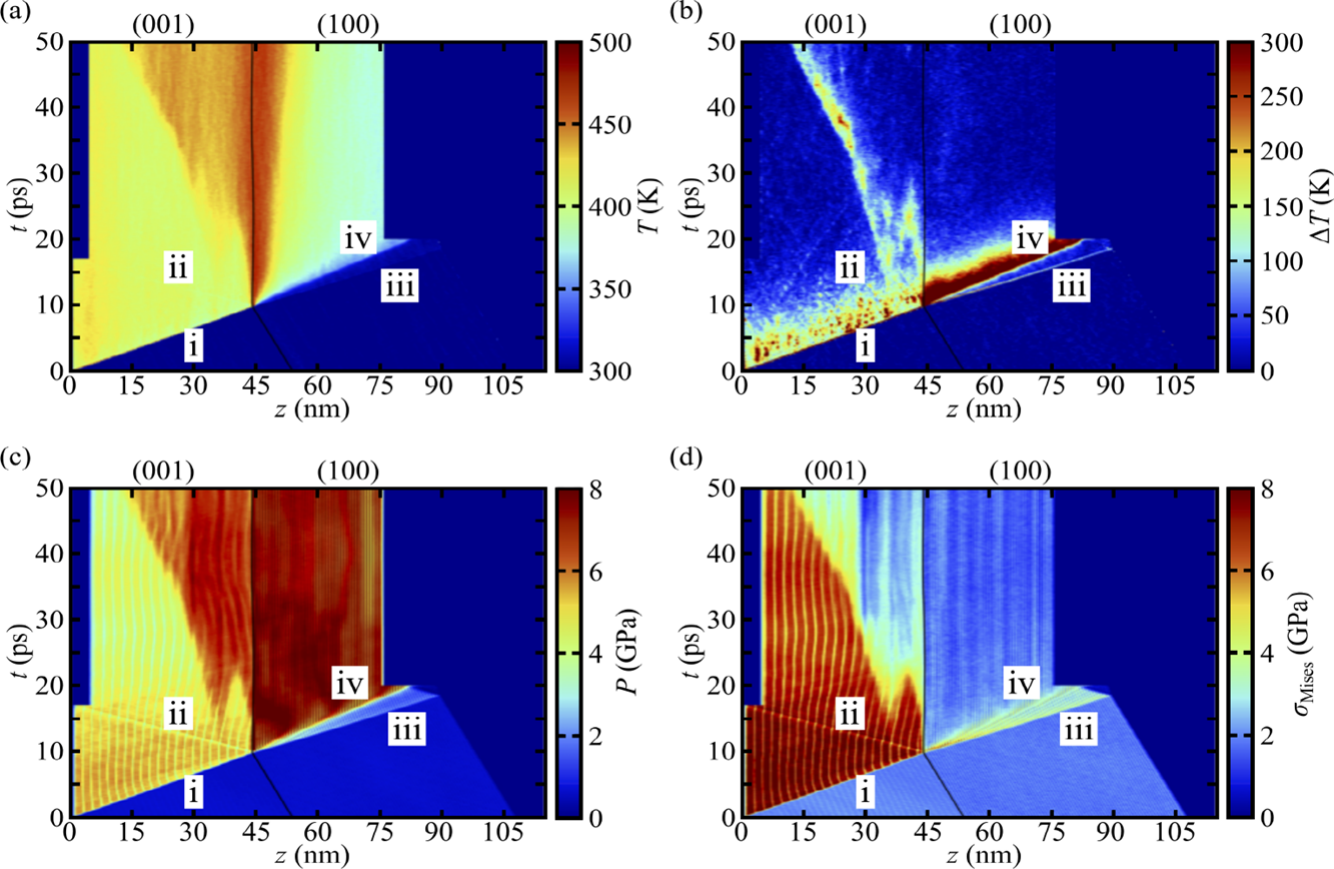
For the +*z* shock case, extended time histories for (a) the kinetic temperature, (b) the difference Δ*T* between kinetic temperatures of the translational and ro-vibrational DOF, (c) the pressure *P*, and (d) the von Mises stress σ_Mises_. The location of the GB is shown as a solid black curve and the wave features previously identified in Figure 5 are indicated by Roman numerals.

Perhaps the most striking feature in Figure 11a is that the hot spot growth is sustained over the entire time interval, ultimately heating most of the (001) grain. There is also growth into the (100) grain, but much less so than in the (001) grain. The final hot spot size is ≈40 nm by the end of the simulation.

As discussed above in connection with Figure 7, hot spot growth in the (001) grain is due to the propagation of shear bands that nucleate at the GB and in the bulk crystal within a short time interval (Δ*t* = 12–15 ps). The propagation of these bands is most clearly seen in the excitation of molecular translational DOF shown in Figure 11b. An inflection point can be seen at *z* ≈ 25 nm and *t* ≈ 30 ps, after which the propagation speed of the shear band increases. Propagating shear bands relieve shear stress and increase the local volumetric stress, which is shown in panels (c) and (d).

While the interfacial hot spot grows in time due to sustained inelastic deformations, it should be emphasized that its formation and growth occur due to separate mechanisms. As identified in Section 3.3, the hot spot forms at the interface due to the concentration of shear stress, which accelerates the layer buckling mechanism in the (100) grain leading to that region undergoing a pseudosingle shock before the wave structure splits. Growth of the hot spot occurs by a completely different inelastic deformation mechanism in the (001) grain, namely through shear band nucleation and propagation.

## 4 Conclusions

Real secondary HEs are typically formulated materials with rich microstructure involving many interfaces with the crystalline energetic constituents. However, the role that these interfaces play in hot spot formation processes that govern detonation initiation remains poorly quantified. To address this gap, we derive extensions for the GCCM to prepare GBs with well-characterized tilt and twist orientations. The extended GCCM tools were used to prepare an MD simulation cell containing an idealized GB between the (001) and (100) crystal facets in the triclinic HE TATB. Shock simulations performed using this computational cell reveal a strong and unanticipated directional dependence to the formation of a hot spot at the GB interface. A hot spot forms at the GB when the shock transits from the (001) grain to the (100) grain, but not vice versa.

We trace the origin of this anisotropy to the disconnect between the time scale for the shock rise to reach an equilibrated temperature state (≈5 ps) versus that for the development of a two-wave structure near the GB interface (≈1 ps). The hot spot regions in our simulations were precisely those that were subject to an unsteady two-wave structure where a secondary wave arrived before the material could fully equilibrate following a primary wave. This leads to a pseudosingle-shock structure that does more work and generates a higher temperature compared to that for a steady two-wave shock structure. In the present case, this disconnect in time scale occurs for physical reasons because the interface accelerates nucleation of inelastic deformations (leading to an initially unsteady secondary wave), which occurs more quickly than intramolecular vibrational coupling can equilibrate the deposited kinetic energy among the molecular DOF (leading to comparatively slow shock-rise time). This microscale phenomenon is entirely physical, but is analogous to overheating due to entropy errors arising from artificial viscosity at doubly shocked interfaces in hydrocode simulations.

In the present case, the temperature of the hot spot formed at the GB is well below the thresholds for prompt (<μs) chemical reactions in TATB. This is not surprising as the shock pressure in our MD simulations was very close to the threshold for detonation initiation failure in TATB-based HE formulations.^72,90^ More extensive characterizations at stronger shock pressures would be required to determine if GB hot spots in TATB might reach temperatures where they could spontaneously ignite. Regardless of whether GB hot spots can ignite in TATB, they do serve to heat the surrounding crystal, which can promote the reactive burning and growth of other hot spots such as those formed from pore collapse. TATB is also a very insensitive HE. It is possible that GB hot spots might reach high-enough temperatures to trigger prompt chemical reactions in other more sensitive HEs.

We anticipate that the extended GCCM tools presented here will help improve the precision of MD constructions used to assess the role of interfaces in HEs and other crystalline molecular materials. While the present study focused on predicting shock responses at interfaces, GCCM constructions can help quantify adhesion between crystal grains,^34,37^ elucidate the role of interfaces as nucleation points for melting,^34^ and predict how interfaces serve as points for mechanical failure under other loading conditions.^91^ Such studies are expected to help improve and constrain the physics incorporated in coarse grain models used for simulations at larger length scales, including models that treat interfaces in an implicit manner.

## Supplementary Material



## Notes

Velocity field components for the two shock simulations (PDF)
