# Supplementary material for: Anisotropic Hot Spot Formation at a Grain Boundary in Shock-Compressed TATB High Explosive Crystal
Source: J Phys Chem C Nanomater Interfaces. 2026 Apr 14;130(16):6005–19. doi: 10.1021/acs.jpcc.6c00496 (PMC13112440; doi:10.1021/acs.jpcc.6c00496)
Supplement: Supplementary file 1 [file jp6c00496_si_001.pdf]

Supporting Information: Anisotropic Hot Spot Formation at a Grain Boundary in Shock-compressed TATB High Explosive Crystal

Matthew P. Kroonblawd,[*,†] Nithin Mathew,[‡] Puhan Zhao,[¶] Shan Jiang,[||] Edward M. Kober,[*,⊥] and Tommy Sewell[*,#]

†*Physical and Life Sciences Directorate, Lawrence Livermore National Laboratory, Livermore, CA 94550, United States*

‡*X Computational Physics Division, Los Alamos National Laboratory, Los Alamos, NM 87545, United States*

¶*Center for Clinical Pharmacology, Washington University School of Medicine and University of Health Sciences and Pharmacy, St. Louis, MO 63110, United States*

§*Department of Anesthesiology, School of Medicine, Washington University in St. Louis, St. Louis, MO 63110, United States*

||*Department of Mechanical Engineering, University of Mississippi, University, MS 38677, United States*

⊥*Theoretical Division, Los Alamos National Laboratory, Los Alamos, NM 87545, United States*

#*Department of Chemistry, University of Missouri, Columbia, MO 65211, United States*

@*Materials Science and Engineering Institute, University of Missouri, Columbia, MO 65211, United States*

E-mail: kroonblawd1@llnl.gov; emk@lanl.gov; sewellt@missouri.edu

1 Supporting Figure

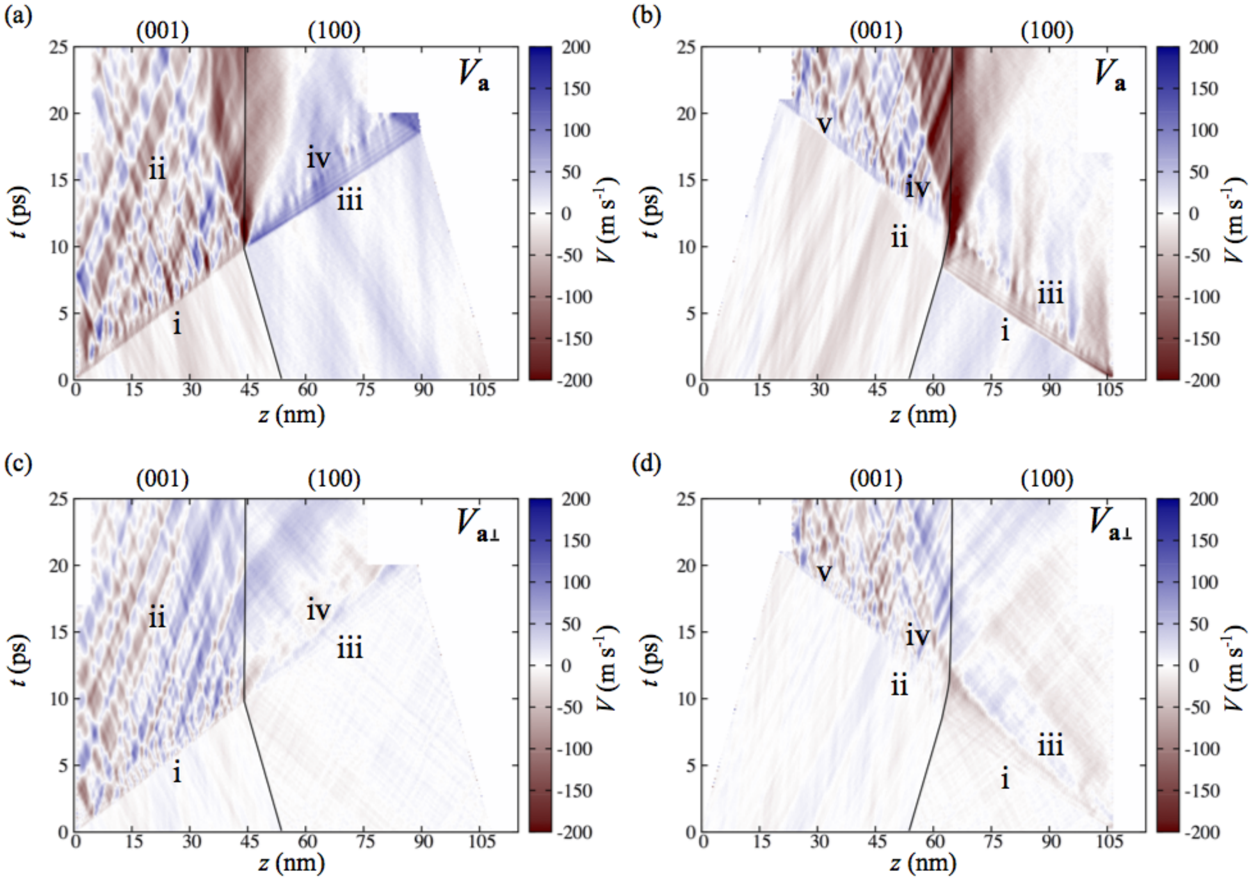

Figure S1: Comparison of the velocity field along two orthogonal directions in the x-y plane, which is transverse to the shock direction z. These directions are chosen to be along lattice vector $\mathbf{a}$ in the (001) grain and the along vector $\mathbf{a}_{\perp}$, which is perpendicular to $\mathbf{a}$ in the (001) grain. The velocity field along $\mathbf{a}$ is shown for the $+z$ and $-z$ shock cases in panels (a) and (b) and correspondingly along $\mathbf{a}_{\perp}$ in panels (c) and (d).

Acknowledgement

EMK and TS thank Ralph Menikoff for many in-depth discussions.

This work was performed under the auspices of the U.S. Department of Energy by Lawrence Livermore National Laboratory under Contract DE-AC52-07NA27344. Approved for unlimited release: LLNL-JRNL-2014638.

This work was supported by the U.S. Department of Energy (DOE) through the Los Alamos National Laboratory. The Los Alamos National Laboratory is operated by Triad National Security, LLC, for the National Nuclear Security Administration of the U.S. Department of Energy (contract no. 89233218CNA000001). Approved for unlimited release: LA-UR-25-28693.

MPK was supported in part by the Laboratory Directed Research and Development Program at Lawrence Livermore National Laboratory; project 24-SI-004 with Kyle Sullivan as P.I. EMK and NM were supported through the ASC program at Los Alamos National Laboratory. PZ, SJ, and TS were supported by the U.S. Air Force Office of Scientific Research (AFOSR); federal award nos. FA9550-13-1-0164 and FA9550-19-1-0318, program officer Martin J. Schmidt. PZ and SJ contributed to this work while at the University of Missouri. The MD simulations were performed using a Linux cluster acquired under the auspices of the U.S. Department of Defense "DURIP" equipment grant program; federal award no. N00014-12-1-0814, Office of Naval Research program officer Dan Prono.
